# Evaluation of the Continuous Positive Airway Pressure Effect on Neurotrophins’ Gene Expression and Protein Levels

**DOI:** 10.3390/ijms242316599

**Published:** 2023-11-22

**Authors:** Agata Gabryelska, Szymon Turkiewicz, Marta Ditmer, Adrian Gajewski, Piotr Białasiewicz, Dominik Strzelecki, Maciej Chałubiński, Marcin Sochal

**Affiliations:** 1 Department of Sleep Medicine and Metabolic Disorders, Medical University of Lodz, 90-419 Lodz, Poland; 2 Department of Immunology and Allergy, Medical University of Lodz, 90-419 Lodz, Poland; 3 Department of Affective and Psychotic Disorders, Medical University of Lodz, 90-419 Lodz, Poland

**Keywords:** CPAP, neurotrophic factors, sleep apnea, biomarkers, intermittent hypoxia

## Abstract

Neurotrophins (NT) might be associated with the pathophysiology of obstructive sleep apnea (OSA) due to concurrent intermittent hypoxia and sleep fragmentation. Such a relationship could have implications for the health and overall well-being of patients; however, the literature on this subject is sparse. This study investigated the alterations in the serum protein concentration and the mRNA expression of the brain-derived neurotrophic factor (BDNF), glial cell line-derived neurotrophic factor (GDNF), neurotrophin-3 (NTF3), and neurotrophin-4 (NTF4) proteins following a single night of continuous positive airway pressure (CPAP) therapy. This study group consisted of 30 patients with OSA. Venous blood was collected twice after a diagnostic polysomnography (PSG) and PSG with CPAP treatment. Gene expression was assessed with a quantitative real-time polymerase chain reaction. An enzyme-linked immunosorbent assay was used to determine the protein concentrations. After CPAP treatment, BDNF, proBDNF, GDNF, and NTF4 protein levels decreased (*p* = 0.002, *p* = 0.003, *p* = 0.047, and *p* = 0.009, respectively), while NTF3 increased (*p* = 0.001). Sleep latency was correlated with ΔPSG + CPAP/PSG gene expression for BDNF (R = 0.387, *p* = 0.038), NTF3 (R = 0.440, *p* = 0.019), and NTF4 (R = 0.424, *p* = 0.025). OSA severity parameters were not associated with protein levels or gene expressions. CPAP therapy could have an impact on the posttranscriptional stages of NT synthesis. The expression of different NTs appears to be connected with sleep architecture but not with OSA severity.

## 1. Introduction

Obstructive sleep apnea (OSA) is a common condition, the main features of which are intermittent hypoxia and sleep fragmentation, caused by the collapse of the airways during sleep [1,2,3]. OSA is treated with continuous positive airway pressure (CPAP) ventilation during the night, which maintains the patency of the upper respiratory tract [4,5]. This condition has a wide range of complications that transcend sleep medicine, such as hypertension, congestive heart disease, immune-mediated conditions, or metabolic syndrome [6,7,8]. During recent decades, the psychiatric and neurocognitive sequelae of OSA, their molecular background, and their prevention have gained the interest of researchers, becoming a promising subject of studies [9,10,11,12]. They include the impairment of memory and executive functions, reduced alertness, and daytime sleepiness, as well as depression and dementia [13,14]. 

Thus, neurotrophins (NTs) have emerged as an important aspect of the OSA pathophysiology. There are four canonical NTs as follows: the nerve growth factor, brain-derived neurotrophic factor (BDNF), neurotrophin-3 (NTF3), and neurotrophin-4 (NTF4). The glial cell line-derived neurotrophic factor (GDNF) is a neurotrophic factor; however, it is functionally similar to the previous proteins [1,15]. NTs are responsible for maintaining the function of the central nervous system and exerting a neuroprotective effect, as well as promoting neurogenesis and gliogenesis [1,16,17]. Currently, most studies focus on BDNF as the most abundant NT; indeed, it seems to be connected to OSA on multiple levels. Shah et al. [18] demonstrated that BDNF expression was upregulated in the muscle fibers of the uvula in this group, which could aid in the recovery of mechanically damaged neurons, supporting the maintenance of the patent airways [18,19,20]. Flores et al. reported that OSA patients tended to exhibit increased peripheral levels of BDNF compared with healthy subjects [21]. Moreover, in their study, this parameter correlated positively with the Montreal Cognitive Assessment score, indicating its connection to cognitive functions [21]. An increased peripheral BDNF level might be a compensatory response to intermittent hypoxia, which is supposed to counteract its degenerative influence on the central nervous system [21,22,23].

The literature on the subject of the connection between other NTs and OSA is sparse. NGF appears to be related to sleep disorders in general; adolescents with excessive daytime sleepiness and poor sleep tend to exhibit lower peripheral levels of this protein [24]. Shah et al. did not detect any differences in its expression in the muscle fibers of the uvula between OSA patients and healthy patients, which indicates that NGF might not be as involved in the recovery of neurons and airway patency as BDNF [18]. However, in children, NGF could be involved in the development of tonsillar hypertrophy [25,26]. GDNF might constitute a part of the genetic background of OSA. In their studies, Larkin et al. demonstrated that certain variants of the GDNF gene are connected to OSA risk in European Americans [27]. Other authors showed that the peripheral GDNF level might be lower in OSA patients compared with healthy controls [28]. 

The subject of the influence of CPAP on NTs also remains underexplored; available studies have mostly analyzed only BDNF serum levels, omitting the other NTs or the expression of genes [1]. Similarly, little is known about NTs in the context of OSA severity parameters or the structure of sleep [1]. GDNF gene variants were associated with the apnea/hypopnea index [27]. 

Thus, this study aimed to analyze the concentration of BDNF, GDNF, NTF3, and NTF4 proteins and the expression of their respective genes, as well as correlate them with polysomnographic parameters.

## 2. Results

The participants included in this study had a median age of 57.00 (46.75–62.25) years old and had a median BMI of 35.11 (31.97–38.37) kg/m^2^; 90.0% (*n* = 27) of the group were men. The median arousal index was 22.30 (14.95–31.20) events/h, the median apnea–hypopnea index (AHI) was 47.95 (24.75–67.20) events/h, and the median desaturation index was 50.60 (27.13–78.65) events/h. In addition, this study group had a median sleep efficiency of 86.20% (74.80–89.70) and a sleep maintenance efficiency of 91.70% (80.00–93.20). Baseline PSG data are shown in Table 1.

There was a decrease in the protein levels of BDNF, proBDNF, GDNF, and NTF4 (*p* = 0.002, *p* = 0.003, *p* = 0.047, and *p* = 0.009, respectively), while NTF3 increased after a PSG with CPAP compared to the initial PSG examination (*p* = 0.001) (Table 2).

Simultaneously, no differences were observed between the gene expression of all the examined neurotrophins between assessed time points (Table 3).

From the demographic parameters only, age was negatively correlated with the BDNF and proBDNF level after PSG with CPAP treatment (R = −0.042, *p* = 0.028 and R = −0.388, *p* = 0.034, respectively).

Sleep efficiency and sleep maintenance efficiency were negatively correlated with the gene expression of all neurotrophins after PSG (R = −0.644, *p* = 0.003 and R = −0.693, *p* = 0.001 for BDNF; R = −0.458, *p* = 0.049 and R = −0.561, *p* = 0.012 for GDNF; R = −0.489, *p* = 0.033 R = −0.440, *p* = 0.040 for NTF3; R = −0.484, *p* = 0.036 R = −0.488, *p* = 0.038 for NTF4, respectively), as well as positively correlated with the difference between BDNF gene expression after PSG with CPAP treatment and after PSG (R = 0.486, *p* = 0.041 and R = 0.631, *p* = 0.005, respectively); no correlation was found between neurotrophin gene expressions after PSG with CPAP treatment with PSG parameters. Furthermore, out of the PSG parameters, sleep onset latency was positively correlated with the difference between gene expression after PSG with CPAP treatment and after PSG for BDNF (R = 0.387, *p* = 0.038), NTF3 (R = 0.440, *p* = 0.019), and NTF4 (R = 0.424, *p* = 0.025).

Out of the PSG variables, only the time and percentage of stage 2 NREM were negatively correlated with protein concentrations including, in particular, proBDNF (R = −0.465, *p* = 0.010 and R = −0.463, *p* = 0.010, respectively) and BDNF (R = −0.379, *p* = 0.039 and R = −0.390, *p* = 0.033, respectively) following PSG as well as positively correlated with the difference between the BDNF protein levels after PSG with CPAP treatment and after PSG (R = 0.398, *p* = 0.029 and R = 0.395, *p* = 0.031, respectively).

Additionally, positive correlations were observed between protein levels at the two evaluated time points for NTF3 (R = 0.720, *p* < 0.001) and NTF4 (R = 0.569, *p* = 0.001). 

Moreover, gene expression was positively correlated with the protein level for NTF3 after PSG with CPAP treatment (R = 0.537, *p* = 0.003) and for the difference between PSG with CPAP treatment and PSG alone (R = 0.595, *p* < 0.001), but was negatively correlated for the GDNF difference between PSG with CPAP treatment and PSG alone (R = −0.397, *p* = 0.033). 

The results described above are presented in Table 4.

## 3. Discussion

The primary finding of this study was that, after a single night of CPAP treatment, the serum protein level of BDNF, proBDNF, GDNF, and NTF4 decreased, while NTF3 increased. This is in line with the available literature; Staats et al. also reported a stark decline in serum BDNF protein after a single night of CPAP treatment, which was maintained after 3 months [29]. It was suggested that the baseline increase in the level of this NT was a compensatory mechanism developed in response to intermittent hypoxia (IH), which is an inherent feature of OSA [21,30]. Little is known about the other NTs in this disorder, but they could fulfill similar protective functions to BDNF [1,31]. Since proBDNF usually exerts the opposite effect to its mature form, its decrease is counterintuitive. However, our previous study indicated its involvement in protection against hypoxia through interactions with hypoxia-inducible factor 1 (HIF-1). Even though a single night of CPAP treatment did not cause changes in HIF-1α levels, it is possible that related proteins react more dynamically to the normalization of SpO_2_ [32]. An increase in the NTF3 protein could be compensatory to a decrease in BDNF, as they cooperate in the prevention of apoptosis [33,34]. 

A negative correlation between the serum BDNF protein concentration after PSG with CPAP treatment and age is consistent with studies conducted up to date and could be perceived as a physiological element of aging [35,36,37]. As for the decrease in proBDNF, the literature on the relationship between this protein and age is limited. In our previous study, we noted a similar pattern of alterations in BDNF and its precursor depending on age [38]. In a study by Li et al., neither BDNF mRNA nor proBDNF was associated with age in healthy subjects; thus, it could be hypothesized that OSA modulates interactions between aging and BDNF synthesis [39]. 

What is interesting is that none of the OSA parameters were correlated with the studied proteins or gene expressions. Studies on the subject vary; Arslan et al. reported that BDNF bore no correlation to AHI or the oxygen desaturation index, whereas Wang observed an association with AHI [40,41]. Kaminska et al. also did not note any relationship between the severity markers of BDNF and OSA [42]. In the case of GDNF, its different genetic variants were demonstrated to have an association with AHI in a population of European Americans [27]. Such results were not replicated in a study in an Icelandic population, where the GDNF gene was not related to OSA [28]. Since this NT is known to influence the development of the respiratory drive, it might be one of the central factors contributing to the hereditary background of OSA, which in certain populations could be more pronounced than in others [1,43].

As for sleep structure, all the NTs were tightly connected to sleep maintenance and efficiency, which gives insight into their relationship to sleep fragmentation, which is another prominent feature of OSA. However, the correlation between BDNF, its precursor, and stage N2 of NREM is unexpected. Studies have usually associated this NT only with N3, emphasizing the importance of this phase in neuroplasticity [44]. Nevertheless, other researchers showed an association between a reduction in BDNF production caused by Val66Met polymorphism and N2 spindles, as well as the influence of N2 on memory [45].

The limitations of this study include a lack of assessment of other variables influencing the level of NTs, like physical activity and the evaluation of short-term CPAP treatment only, as well as the small number of study participants.

To summarize, even a single night of CPAP treatment affects the posttranscriptional stages of synthesis of NTs in patients with OSA. The interactions between sleep changes in OSA and NTs in the context of neuroplasticity, among others, should be further studied.

## 4. Materials and Methods

This study group consisted of 30 patients who were diagnosed with OSA after following a nocturnal PSG examination at the Sleep and Respiratory Disorders Centre in Lodz (Poland) and who underwent one night of effective CPAP treatment with PSG monitoring. The inclusion criteria for this study were: aged 18–75 years and a body mass index (BMI) of 20–45 kg/m^2^. The exclusion criteria were as follows: a diagnosed immune-mediated/inflammatory condition (e.g., connective tissue), cancer (active or in medical history), an infection within a month preceding PSG, a chronic respiratory disease (e.g., bronchial asthma), neurological conditions, psychiatric disorders, insomnia in particular, and the use of medications affecting sleep (e.g., benzodiazepines, Z-drugs). The Ethics Committee of the Medical University of Lodz approved this study protocol (RNN/432/18/KE). Written informed consent was obtained from every participant in this study.

### 4.1. Polysomnography and CPAP Treatment 

**Figure 1:**
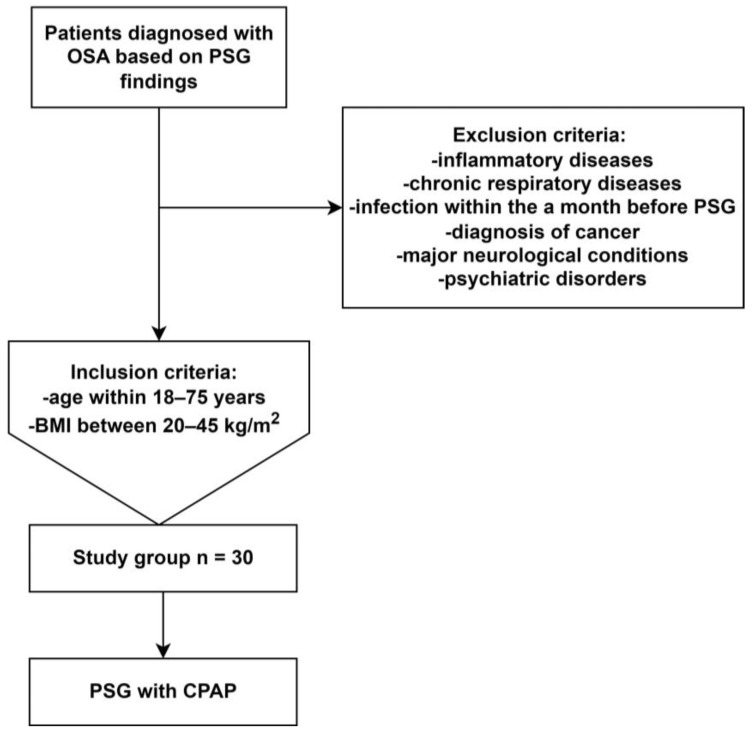
Process of patient selection. Abbreviations: BMI—body mass index, CPAP—continuous positive airway pressure, OSA—obstructive sleep apnea, PSG—polysomnography.

Following admission to the Department at 21:00 h (±0.5 h), patients underwent a physical examination, which included an assessment of their heart rate, blood pressure, body weight, and height. The parameters evaluated during PSG comprised the electroencephalography (C4\A1, C3\A2), respiratory effort (chest and abdomen) and oronasal respiratory airflow measured using a thermistor gauge, electrooculography, electromyography (electrodes placed on the chin and lower limbs), a microphone (snoring detection), body position, electrocardiogram (precordial leads V1 and V2), and the blood oxygen saturation (Alice 6, Phillips-Respironics). The interpretation of PSG was conducted according to the American Academy of Sleep Medicine guidelines using a 30 s epoch standard [46]. The same setup and guidelines were used to monitor CPAP treatment overnight. A flowchart depicting patient selection is depicted in Figure 1.

### 4.2. Assessment of Protein and mRNA Level

Peripheral blood samples were collected in the morning following PSG and PSG with CPAP treatment and an examination into the collection tubes with a clot activator and EDTA (06:00–07:00 h, within 10 min of awakening). Blood samples with a clot activator were centrifuged immediately following blood draws at 4 °C. Serum was collected and stored at −80 °C. The serum neurotrophin protein concentration was assessed using an ELISA kit (FineTest for BDNF and proBDNF, EIAab Science for GDNF, NTF3, and NT4 (Wuhan, China)). The absorbance was measured at λ = 450 nm wavelength using an absorbance reader (BioTek 800 TS, Agilent Technologies, Santa Clara, CA, USA). RNA isolation from peripheral blood leukocytes was performed using the TRIzol reagent (Invitrogen, Waltham, MA, USA). The RNA Integrity Number (RIN), as well as the concentration of the isolated RNA, was assessed using a Nanodrop Colibri Microvolume Spectrometer (Titertek Berthold, Bad Wildbad, Germany). The obtained material was reversely transcribed using a dedicated kit and according to the protocol provided by the manufacturer (SuperScript IV First-Strand Synthesis System, Thermo Fisher Scientific Inc., San Jose, CA, USA). The process comprised 3 steps, and the assays underwent annealing at 60 °C in 60 s. The level of expression of the chosen genes was determined using a quantitative real-time polymerase chain reaction; the applied mixture consisted of nuclease-free water, Master Mix TaqMan Universal, cDNA, and gene-specific probes (TaqMan assays for BDNF, GDNF, NTF3, and NTF4; reference gene: β-Actin). Three reactions were performed for each sample and the reference gene. For each sample, the cycle threshold (CT) was calculated. Then, ∆Ct was calculated and used in the mRNA expression analysis in accordance with the following Equation 2^−∆Ct^ [47].

### 4.3. Statistical Analysis

*p* < 0.05 was considered statistically significant. Data analysis was conducted with the use of SPSS 28.0 (IBM, Chicago, IL, USA). Data distribution was assessed using the Shapiro–Wilk test. The parameters with normal distribution were compared using a paired *t*-test; otherwise, the Wilcoxon test was used to compare the dependent variables. Normally distributed data are presented as the mean ± standard deviation or median and interquartile range (IQR) to allow for comparison with other variables, while parameters with non-normal distribution are presented as the median and IQ. Spearman’s rank correlation was used to assess correlations. For multiple tests, the Bonferroni correction was applied.

## Figures and Tables

**Table 1:** Baseline polysomnographic characteristics of participants.

PSG Parameter	Median (IQR)
Sleep Efficiency [%]	86.20 (74.80–89.70)
Sleep Maintenance [%]	91.70 (80.00–93.20)
Sleep Onset Latency [min]	16.00 (9.00–27.00)
Stage 1 nREM [h]	2.10 (1.65–3.41)
Stage 2 nREM [h]	1.94 (1.07–2.78)
Stage 3 nREM [h]	0.56 (0.13–1.14)
TST [h]	6.55 (5.75–7.24)
REM [h]	1.17 (0.72–1.43)
nREM [h]	5.31 (4.88–5.81)
Arousal Index [events/h]	22.30 (14.95–31.20)
AHI [events/h]	47.95 (24.75–67.20)
Desaturation Index [events/h]	50.60 (27.13–78.65)
Total Number of Desaturations	303.00 (137.75–349.50)
Minimum Oxygen Saturation [%]	71.40 (64.95–76.00)

Abbreviations: AHI—apnea–hypopnea index; IQR—interquartile range; PSG—polysomnography; nREM—non-rapid eye movement; REM—rapid eye movement; TST—total sleep time.

**Table 2:** Comparison of protein levels after a diagnostic polysomnography and a single night of continuous positive airway pressure treatment.

		Time Point		Difference between after PSG with CPAP and after PSG	Increase from after PSG to PSG with CPAP (*n*; %)	*p*-Value
		After PSG	After PSG with CPAP			
Proteins	BDNF [ng/mL]	14.80 (10.40–20.61)	6.53 (2.96–12.80)	−12.63 ((−12.63)–2.32)	9 (30.00%)	**0.002**
	proBDNF [ng/mL]	6.27 (4.95–8.76)	3.31 (1.62–5.26)	−3.10 ((−5.83)–1.19)	10 (33.33%)	**0.003**
	GDNF [ng/mL]	96.91 (85.93–129.38)	92.47 (82.89–97.14)	0.00 ((−35.16)–7.13)	15 (50.00%)	**0.047**
	NTF3 [ng/mL]	148.43 (130.23–172.96)	169.00 (140.04–219.16)	18.04 (1.74–50.16)	23 (76.67%)	**0.001**
	NTF4 [pg/mL]	2.35 (1.80–3.26)	1.78 (0.80–2.48)	−0.53 ((−1.13)–0.14)	8 (26.67%)	**0.009**

Abbreviations: BDNF—brain-derived neurotrophic factor, GDNF—glial cell line-derived neurotrophic factor, NFT3—neurotrophin-3, NFT4—neurotrophin-4. Data are presented as median and interquartile range (IQR). Bold text indicated statistical significance.

**Table 3:** Comparison of gene expressions after a diagnostic polysomnography and a single night of continuous positive airway pressure treatment.

		Time Point		Difference between after PSG with CPAP and after PSG	Increase from after PSG to PSG with CPAP (*n*; %)	*p*-Value
		After PSG	After PSG with CPAP			
Gene Expression (ΔCt) × 100	BDNF	19.20 (6.02–84.38)	72.32 (30.27–107.23)	8.72 ((−46.00)–82.97)	17 (56.67%)	0.338
	GDNF	10.16 (0.97–41.08)	26.21 (17.26–39.91)	17.56 ((−25.20–29.23)	17 (56.67%)	0.432
	NTF3	18.89 (3.07–87.56)	39.96 (20.55–80.81)	9.13 ((−53.40)–43.34)	16 (53.33%)	0.347
	NTF4	33.35 (1.16–17.37)	69.29 (34.72–246.63)	18.30 ((−93.10)–163.58)	18 (60.00%)	0.622

Abbreviations: BDNF—brain-derived neurotrophic factor, GDNF—glial cell line-derived neurotrophic factor, NFT3—neurotrophin-3, NFT4—neurotrophin-4. Data are presented as median and interquartile range (IQR).

**Table 4:** Correlations between neurotrophins, selected sleep, and molecular parameters.

		R	*p*
Sleep Efficiency	BDNF mRNA after PSG	−0.644	0.003
	GDNF mRNA after PSG	−0.458	0.049
	NTF3 mRNA after PSG	−0.489	0.033
	NTF4 mRNA after PSG	−0.484	0.036
	Difference between BDNF mRNA expression after PSG with CPAP and after PSG	0.486	0.041
Sleep Maintenance Efficiency	BDNF mRNA after PSG	−0.693	0.001
	GDNF mRNA after PSG	−0.561	0.012
	NTF3 mRNA after PSG	−0.440	0.040
	NTF4 mRNA after PSG	−0.488	0.038
	Difference between BDNF mRNA expression after PSG with CPAP and after PSG	0.631	0.005
Sleep Onset Latency	Difference between BDNF mRNA expression after PSG with CPAP and after PSG	0.387	0.038
	Difference between NTF3 mRNA expression after PSG with CPAP and after PSG	0.440	0.019
	Difference between NTF4 mRNA expression after PSG with CPAP and after PSG	0.424	0.025
Stage 2 NREM duration	proBDNF serum protein concentration	−0.465	0.010
	BDNF serum protein concentration	−0.379	0.039
	Difference between BDNF serum protein concentration after PSG with CPAP and after PSG	0.398	0.029
Stage 2 NREM percentage	proBDNF serum protein concentration	−0.463	0.010
	BDNF serum protein concentration	−0.390	0.033
	Difference between BDNF serum protein concentration after PSG with CPAP and after PSG	0.395	0.031
NTF3 serum protein concentrations after PSG with CPAP and after PSG		0.720	<0.001
NTF4 serum protein concentrations after PSG with CPAP and after PSG		0.569	0.001
NTF3 mRNA and serum protein concentration after PSG with CPAP		0.537	0.003
NTF3 mRNA and the difference between NTF3 serum protein concentration after PSG with CPAP and after PSG		0.595	<0.001
GDNF mRNA and the difference between GDNF serum protein concentration after PSG with CPAP and after PSG		−0.397	0.033

Abbreviations: BDNF—brain-derived neurotrophic factor, GDNF—glial cell line-derived neurotrophic factor, NFT3—neurotrophin-3, NFT4—neurotrophin-4, NREM—Non-rapid eye movement sleep.

## Data Availability

Data will be made available upon request.
